# Is Neoadjuvant Chemotherapy Necessary for Male Breast Cancer? A SEER‐Based Population Study

**DOI:** 10.1155/tbj/3718304

**Published:** 2026-09-16

**Authors:** Liwei Pang, YiFan Li, Zhen Wang

**Affiliations:** ^1^ Department of Breast Surgery, Second Affiliated Hospital, Zhejiang University School of Medicine, Hangzhou 310009, China, zju.edu.cn; ^2^ Key Laboratory of Tumor Microenvironment and Immune Therapy of Zhejiang Province, Second Affiliated Hospital, Zhejiang University School of Medicine, Hangzhou 310009, China, zju.edu.cn

**Keywords:** male breast cancer, neoadjuvant chemotherapy, overall survival, pathologic complete response

## Abstract

**Background and Aims:**

Neoadjuvant chemotherapy (NAC) is widely utilized in female breast cancer to achieve disease downstaging, reduce distant dissemination, and assess chemosensitivity. However, the efficacy of NAC in male breast cancer (MBC) remains poorly understood. This study aimed to evaluate pathologic complete response (pCR) rates and overall survival (OS) outcomes following NAC in MBC patients, stratified by tumor subtype.

**Methods:**

Data were extracted from the Surveillance, Epidemiology, and End Results (SEER) database for MBC patients who received NAC between 2000 and 2020. Patients were categorized by receptor status: hormone receptor–positive/human epidermal growth factor receptor 2–negative (HR+/HER2−), HR+/HER2+, HR−/HER2+, and HR−/HER2−. The proportions of pCR (ypT0/Tis ypN0) were compared across subtypes. OS was estimated using the Kaplan–Meier method, and differences were analyzed using the log‐rank test. Propensity score matching (PSM) was applied to reduce selection bias inherent to this retrospective observational study. Based on the baseline characteristics of breast cancer patients who received NAC, univariate Cox proportional hazards regression analysis was first performed to screen potential prognostic variables. Covariates with statistically significant differences in the univariate model were subsequently incorporated into the multivariate Cox regression model to identify independent risk factors associated with OS prognosis. Statistical analyses were performed using STATA 12.0 and SPSS Version 23.0.

**Results:**

A total of 260 MBC patients who received NAC were included in the analysis. No statistically significant difference in OS was observed between MBC and FBC patients receiving NAC (HR = 1.067; 95% CI: 0.929–1.226; *p* = 0.358). However, female patients had a better OS (HR = 0.930; 95% CI: 0.913–0.948; *p* < 0.001) after PSM. Five‐year OS for MBC patients with pCR versus non‐pCR was 92% vs. 77.3% (HR = 1.236; 95% CI: 0.787–1.942; *p* = 0.357). The results were further confirmed in the multivariate Cox regression model after PSM, in which pCR can be considered an independent prognostic factor. In addition, adjuvant chemotherapy demonstrated superior OS compared to NAC in MBC patients (HR = 3.223; 95% CI: 2.772–3.747; *p* < 0.001). The proportions of pCR by tumor subtype were as follows: (i) HR−/HER2+: 50.0% (*n* = 3), (ii) HR−/HER2−: 41.7% (*n* = 5), (iii) HR+/HER2+: 10.7% (*n* = 7), and (iv) HR+/HER2−: 5.5% (*n* = 9). OS was significantly worse with NAC compared to adjuvant chemotherapy in both HR+/HER2− (HR = 1.363; 95% CI: 1.129–1.646; *p* = 0.001) and HR+/HER2+ (HR = 1.951; 95% CI: 1.449–2.627; *p* < 0.001) subtypes. However, no statistically significant differences were observed in HR−/HER2+ (HR = 2.296; 95% CI: 0.807–6.538; *p* = 0.119) and HR−/HER2− (HR = 1.758; 95% CI: 0.847–3.649; *p* = 0.130).

**Conclusion:**

This population‐based study demonstrates that MBC patients receiving NAC achieved significantly lower pCR rates compared to female patients. The HR+/HER2− and HR+/HER2+ subtypes exhibited greater resistance to NAC. Furthermore, achieving pCR was not prognostic for improved survival in MBC. These findings suggest that NAC may not be necessary for all MBC cases. Future research should explore alternative treatment strategies tailored to the unique biological characteristics of MBC.

## 1. Introduction

Male breast cancer (MBC) is a rare malignancy, representing approximately 1% of all cancers diagnosed in men and approximately 1% of all breast cancers worldwide. Due to its rarity, the pathophysiology of MBC remains poorly characterized, and treatment recommendations are typically extrapolated from clinical trials conducted in female breast cancer patients [1]. Neoadjuvant chemotherapy (NAC) is increasingly utilized in the preoperative setting for breast cancer to achieve disease downstaging, reduce distant metastases, and evaluate chemosensitivity [2]. Current clinical guidelines recommend that men with breast cancer follow the same NAC and anti–human epidermal growth factor receptor 2 (HER2) treatment protocols as women [3, 4]. Accurate assessment of response to NAC provides critical insights into the impact of systemic therapies on breast cancer biology, prognosis, and guidance for subsequent therapy, with pathologic complete response (pCR) serving as a key efficacy measure for NAC [5]. However, the efficacy of NAC in MBC and its clinical outcomes remain largely unknown.

To better understand the efficacy of NAC in MBC, we analyzed data from the National Cancer Institute’s (NCI) Surveillance, Epidemiology, and End Results (SEER) database for MBC patients who received NAC. We examined the proportion of pCR by tumor subtype, patient characteristics, and overall survival (OS) outcomes.

## 2. Materials and Methods

### 2.1. Data Source and Study Design

Data for this study were obtained from the SEER program, which collects cancer incidence, prevalence, and survival data from US cancer registries. The database undergoes rigorous quality control and includes comprehensive tumor characteristics, clinical and pathological staging, treatment information, and survival outcomes.

**FIGURE 1:**
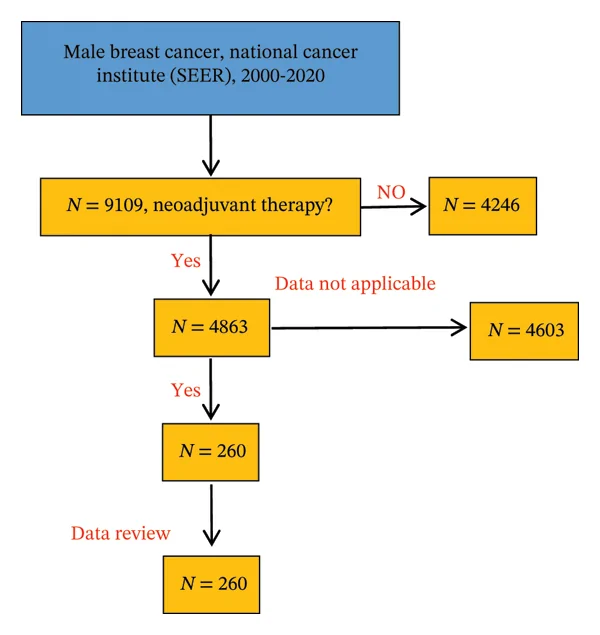
The flow diagram of the patient population included in the study.

Using SEER data, we identified MBC patients treated with NAC between January 1, 2000, and December 31, 2020. Patients were excluded if information regarding hormone receptor (HR) status, HER2 status, clinical TNM staging, or response to NAC was unavailable. Figure 1 presents a flow diagram of the patient selection process.

Patients were categorized based on receptor status as follows: HR‐positive (HR+)/HER2‐negative (HER2−), HR+/HER2+, HR−/HER2+, and HR−/HER2−. Pathologic response and OS were evaluated for each subgroup.

### 2.2. Outcomes

The primary endpoint was pCR, defined as the absence of invasive cancer in both the breast and axillary lymph nodes, regardless of the presence of ductal carcinoma in situ (ypT0/Tis, ypN0). We compared the proportion of patients achieving pCR for each tumor subtype in MBC.

Secondary endpoints included clinical response and OS. Clinical response data were obtained from the SEER variable “Response to Neoadjuvant Therapy Recode,” which categorizes responses as complete response, partial response, response to treatment (not specified as complete or partial), or no response. OS was defined as the time from breast cancer diagnosis to death from any cause or last follow‐up for censored patients.

### 2.3. Statistical Analysis

Chi‐square test was used to compare baseline characteristics between patients. OS was estimated using the Kaplan–Meier method and compared by tumor subtype and pCR status using the log‐rank test. Multivariate prognostic analysis was subsequently performed using the Cox proportional hazards regression model. To eliminate the confounding effects of covariates, propensity score matching (PSM) with a 1:1 matching ratio was conducted based on the following variables: age, race, pathological T stage, pathological N stage, pathological M stage, overall stage, and subtype. The differences in OS and baseline covariates between the two groups before and after PSM were re‐compared to verify the robustness of the analytical results. All *p* values were two‐sided, and *p* < 0.05 was considered statistically significant. Data are presented as mean ± standard deviation. Statistical analyses were performed using STATA 12.0 (Stata Corporation, College Station, TX) and SPSS Version 23.0 (SPSS Inc., Chicago, IL).

## 3. Results

### 3.1. Patient Characteristics

Among 9109 MBC cases, 260 patients underwent NAC and were included in the study (see Figure 1). A total of 57,707 women who received NAC and 3136 men who received adjuvant chemotherapy had available survival and vital status information and were included in OS analyses. Baseline characteristics of patients who received NAC are summarized in Table 1. Statistically significant intergender differences were observed in age, race, pathological T stage, pathological N stage, pathological M stage, overall stage, and subtype. MBC patients were older (*p* < 0.001) and presented with more advanced pathological stages (*p* = 0.043). After PSM, a total of 154 patients were successfully matched, including 77 male patients and 77 female patients in each group (Table 2). Statistical analyses revealed that no statistically significant differences were found in pathological T stage, pathological M stage, and overall stage between the two groups after PSM, whereas significant intergroup differences existed in age (*p* = 0.002) and molecular subtype (*p* < 0.001). Among male patients, 164 (62.8%) had HR+/HER2− tumors, 65 (24.9%) had HR+/HER2+ tumors, 6 (2.3%) had HR−/HER2+ tumors, 12 (4.6%) had HR−/HER2− tumors, and 13 patients had unknown receptor status.

**TABLE 1:** Characteristics of participants before PSM.

	Male (*n* = 260)	Female (*n* = 57,707)	Overall (57,967)	*p* value
Age, *n* (%)				< 0.001
< 40	12 (4.6)	8125 (14.1)	8137 (14.0)	
40–64	141 (54.2)	36,813 (63.8)	36,954 (63.8)	
> 64	107 (41.2)	12,769 (22.1)	12,876 (22.2)	
Race, *n* (%)				< 0.001
White	159 (61.2)	32,494 (57.2)	32,653 (57.2)	
Black	65 (25.0)	8603 (15.2)	8668 (15.2)	
Indian	2 (0.8)	424 (0.7)	426 (0.7)	
Asian	17 (6.5)	6059 (10.7)	6076 (10.7)	
Hispanic	15 (5.8)	9016 (15.9)	9031 (15.8)	
Unknown	2 (0.8)	185 (0.3)	187 (0.3)	
Pathological T stage, *n* (%)				< 0.001
T0	0 (0)	89 (0.4)	89 (0.4)	
T1	16 (18.4)	4065 (18.3)	4081 (18.3)	
T2	31 (35.6)	9918 (44.7)	9949 (44.7)	
T3	11 (12.6)	4024 (18.1)	4035 (18.1)	
T4	16 (12.4)	16 (18.4)	2756 (12.4)	
Tis	0 (0.0)	179 (0.8)	179 (0.8)	
Tx	2 (2.3)	458 (2.1)	460 (2.1)	
Any T mets	11 (12.6)	698 (3.1)	709 (3.2)	
Pathological N stage, *n* (%)				0.046
N0	22 (25.3)	7765 (34.0)	7787 (34.0)	
N1	34 (39.1)	9517 (41.7)	9551 (41.6)	
N2	18 (20.7)	2713 (11.9)	2731 (11.9)	
N3	12 (13.8)	2266 (9.9)	2278 (9.9)	
Nx	1 (1.1)	586 (2.6)	287 (2.6)	
Pathological M stage, *n* (%)				0.031
M0	76 (87.4)	21,394 (93.6)	21,470 (93.6)	
M1	11 (12.6)	1376 (6.0)	1397 (6.0)	
Mx	0 (0.0)	77 (0.3)	77 (0.3)	
Stage, *n* (%)				0.043
I	8 (9.2)	2025 (9.0)	2033 (9.0)	
IIA	12 (13.8)	5672 (25.2)	5684 (25.2)	
IIA	17 (19.5)	5339 (23.8)	5356 (23.7)	
IIIA	15 (17.2)	3814 (17.0)	3829 (17.0)	
IIIB	15 (17.2)	2152 (9.6)	2167 (9.6)	
IIIC	7 (8.0)	1603 (7.1)	1610 (7.1)	
IIINOS	0 (0.0)	57 (0.3)	57 (0.3)	
IV	12 (13.8)	1637 (7.3)	1649 (7.3)	
UNK stage	1 (1.1)	176 (0.8)	177 (0.8)	
Subtype, *n* (%)				< 0.001
HR+/HER2+	65 (26.3)	12,955 (23.1)	13,020 (23.1)	
HR+/HER2−	164 (66.4)	22,815 (40.7)	22,979 (40.8)	
HR−/HER2+	6 (2.4)	6441 (11.5)	6447 (11.4)	
HR−/HER2−	12 (4.9)	13,905 (24.8)	13,917 (24.7)	
Clinical response, *n* (%)				< 0.001
CR	25 (9.6)	16,896 (29.3)		
PR	99 (38.1)	18,872 (32.7)		
NR	49 (18.8)	5225 (9.1)		
No PRCR	87 (33.5)	16,714 (29.0)		

**TABLE 2:** Characteristics of participants after PSM.

	Male (*n* = 77)	Female (*n* = 77)	Overall (154)	*p* value
Age, *n* (%)				0.002
< 40	2 (2.6)	8 (10.4)	10 (6.5)	
40–64	43 (55.8)	5 (71.4)	98 (63.6)	
> 64	32 (41.6)	14 (18.2)	46 (29.9)	
Race, *n* (%)				0.008
White	49 (63.6)	38 (49.4)	87 (56.5)	
Black	18 (23.4)	11 (14.3)	29 (188)	
Indian	1 (1.3)	0 (0.0)	1 (0.6)	
Asian	4 (5.2)	13 (16.9)	17 (11.0)	
Hispanic	5 (6.5)	15 (19.5)	20 (13.0)	
Pathological T stage, *n* (%)				0.505
T0	0 (0.0)	1 (1.3)	1 (0.6)	
T1	16 (20.8)	15 (19.5)	31 (20.1)	
T2	26 (33.8)	30 (39.0)	56 (36.4)	
T3	10 (13.0)	15 (19.5)	25 (16.2)	
T4	15 (19.5)	10 (13.0)	25 (16.2)	
Any T mets	6 (7.8)	6 (78)	16 (10.4)	
Pathological N stage, *n* (%)				0.027
N0	20 (26.0)	34 (44.2)	54 (35.1)	
N1	29 (37.7)	30 (39.0)	59 (38.3)	
N2	16 (20.8)	7 (9.1)	23 (14.9)	
N3	12 (15.6)	6 (7.8)	18 (11.7)	
Pathological M stage, *n* (%)				0.291
M0	67 (87.0)	71 (92.2)	138 (89.6)	
M1	10 (13.0)	6 (7.8)	16 (10.4)	
Stage, *n* (%)				0.156
I	8 (10.4)	8 (10.4)	16 (10.4)	
IIA	11 (14.3)	22 (28.6)	33 (21.4)	
IIB	12 (15.6)	18 (23.4)	30 (19.5)	
IIIA	15 (19.5)	10 (13.0)	25 (16.2)	
IIIB	13 (16.9)	10 (13.0)	23 (14.9)	
IIIC	7 (9.1)	3 (3.9)	10 (6.5)	
IV	11 (14.3)	6 (7.8)	17 (11.0)	
Subtype, *n* (%)				< 0.001
HR+/HER2+	17 (22.1)	24 (31.2)	41 (26.6)	
HR+/HER2−	56 (72.7)	18 (23.4)	74 (48.1)	
HR−/HER2+	2 (2.6)	11 (14.3)	13 (8.4)	
HR−/HER2−	2 (2.6)	24 (31.2)	26 (16.9)	
Clinical response, *n* (%)				< 0.001
CR	3 (3.9)	77 (100)		
PR	74 (96.1)	0 (0.0)		
NR	0 (0.0)	0 (0.0)		
No PRCR	0 (0.0)	0 (0.0)		

### 3.2. Pathologic Response to NAC

The overall pCR rate in MBC was relatively low (*n* = 25, 9.6%); however, NAC demonstrated efficacy in specific breast cancer subtypes. The pCR rate for HR−/HER2+ breast cancer in men was 50.0% (*n* = 3), and for HR−/HER2− (triple‐negative) breast cancer, it was 41.7% (*n* = 5). Among HR+/HER2+ breast cancer patients, the pCR rate in men was 10.7% (*n* = 7). The lowest pCR rate was observed in the HR+/HER2− subgroup (5.5%, *n* = 9), which also represented the largest patient cohort. One patient achieved pCR without a reported breast cancer subtype in the SEER database.

### 3.3. Survival Analysis

**FIGURE 2:**
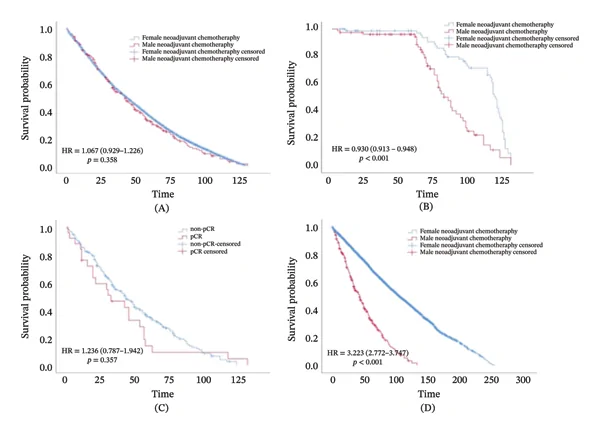
Comparison of OS among different populations. (A) Male neoadjuvant chemotherapy vs. female neoadjuvant chemotherapy before PSM. (B) Male neoadjuvant chemotherapy vs. female neoadjuvant chemotherapy after PSM. (C) pCR vs non‐pCR in male neoadjuvant chemotherapy. (D) Male adjuvant chemotherapy vs. male neoadjuvant chemotherapy.

As a secondary endpoint, we evaluated the associations between OS, pCR status, and tumor subtype. No statistically significant difference in OS was observed between FBC and MBC patients treated with NAC (HR = 1.067; 95% CI: 0.929–1.226; *p* = 0.358) before PSM (Figure 2A). Figure 2B showed a better OS among female breast cancer patients after PSM (HR = 0.930; 95% CI: 0.913–0.948; *p* < 0.001). Furthermore, Figure 2C demonstrates that pCR was not significantly associated with OS in male patients receiving NAC (HR = 1.236; 95% CI: 0.787–1.942; *p* = 0.357). In contrast, adjuvant chemotherapy for MBC demonstrated superior OS compared to NAC (HR = 3.223; 95% CI: 2.772–3.747; *p* < 0.001) (Figure 2D).

### 3.4. Univariate and Multivariate Analyses Based on the Cox Hazard Ratio Model

We performed univariate and multivariate Cox proportional hazards regression analyses both before and after PSM. Univariate Cox regression (Table 3) revealed that age, pCR status, race, pathological T/N/M stage, AJCC overall stage, and molecular subtype were significantly correlated with OS in the pre‐PSM cohort. Notably, sex showed no prognostic significance before matching but emerged as a significant adverse prognostic indicator in the post‐PSM univariate analysis (HR = 2.785; 95% CI: 1.798–4.314; *p* < 0.001). The multivariate Cox model for the pre‐PSM cohort (Table 4) further validated that age, race, overall stage, and molecular subtype acted as independent poor prognostic factors after mutual covariate adjustment. After eliminating baseline imbalance and selection bias via PSM (Table 5), only pCR status remained as an independent adverse prognostic factor for OS (HR = 7.684; 95% CI: 1.653–35.707; *p* = 0.009).

**TABLE 3:** Univariate Cox regression analysis.

	Before PSM HR (95% CI)	*p* value	After PSM HR (95% CI)	*p* value
Male vs. female	1.067 (0.929–1.226)	0.358	2.785 (1.798–4.314)	< 0.001
pCR vs. no pCR	0.930 (0.913–0.948)	**< 0.001**	3.992 (2.381–6.461)	< 0.001
Age		**< 0.001**		0.826
40‐64 vs. < 40	0.908 (0.881–0.936)	< 0.001	0.864 (0.369–2.021)	0.736
> 64 vs. < 40	0.898 (0.878–0.918)	< 0.001	1.082 (0.680–1.7211)	0.739
Race		**< 0.001**		0.510
Black vs. White	0.662 (0.572–0.766)	< 0.001	0.722 (0.397–1.313)	0.286
Indian vs. White	0.705 (0.609–0.817)	< 0.001	1.011 (0.5011–2.039)	0.977
Asian vs. White	0.670 (0.561–0.801)	< 0.001	< 0.001	0.976
Hispanic vs. White	0.766 (03661–0.887)	< 0.001	1.154 (0.497–2.681)	0.738
Unknown vs. White	0.761 (0.657–0.881)	< 0.001		
Pathological T stage		**< 0.001**		0.956
T1 vs. T0	1.430 (1.097–1.865)	< 0.001	< 0.001	0.982
T2 vs. T0	1.556 (1.377–1.758)	0.008	1.297 (0.488–3.450)	0.602
T3 vs. T0	1.493 (1.325–1.683)	< 0.001	1.425 (0.559–3.630)	0.458
T4 vs. T0	1.353 (1.197–1.531)	< 0.001	1.338 (0.497–3.600)	0.564
Tis vs. T0	1.215 (1.069–1.381)	< 0.001		
Tx vs. T0	1.056 (0.851–1.310)	0.003		
Any T mets vs. T0	1.481 (1.266–1.732)	0.620	1.643 (0.584–4.625)	0.347
Pathological N stage		**< 0.001**		0.046
N1 vs. N0	1.114 (1.011–1.227)	0.030	1.053 (0.471–2.357)	0.900
N2 vs. N0	1.034 (0.939–1.139)	0.499	1.306 (0.582–2.928)	0.517
N3 vs. N0	0.932 (0.839–1.036)	0.193	2.574 (1.007–6.580)	0.048
Nx vs. N0	0.963 (0.963–0.861)	0.504		
Pathological M stage		**< 0.001**		0.468
M1 vs. M0	1.456 (1.075–1.971)	0.015	0.716 (0.290–1.770)	0.470
Mx vs. M0	1.217 (0.888–1.667)	0.223		
Stage		**< 0.001**		0.378
IIA vs. I	0.853 (0.708–1.027)	0.093	1.372 (0.475–3.963)	0.559
IIB vs. I	0.823 (0.686–0.988)	0.037	1.027 (0.391–2.699)	0.957
IIIA vs. I	0.763 (0.635–0.916)	0.004	1.705 (0.639–4.550)	0.286
IIIB vs. I	0.699 (0.581–0.840)	< 0.001	1.615 (0.599–4.352)	0.344
IIIC vs. I	0.634 (0.525–0.765)	< 0.001	1.674 (0.587–4.773)	0.335
IIINOS vs. I	0.725 (0.597–0.879)	0.001		
IV vs. I	0.631 (0.425–0.937)	0.022	2.768 (0.779–9.836)	0.115
UNK stage vs. I	0.634 (0.521–0.773)	< 0.001		
Subtype		**< 0.001**		0.055
HR+/HER2− vs. HR+/HER2+	1.027 (1.001–1.054)	0.043	2.200 (1.211–3.995)	0.010
HR−/HER2+ vs. HR+/HER2+	0.895 (0.874–0.916)	< 0.001	1.908 (1.078–3.375)	0.026
HR−/HER2− vs. HR+/HER2+	0947 (0.917–0.977)	0.001	1.485 (0.700–3.153)	0.303

**TABLE 4:** Multivariate cox regression analysis before PSM.

	HR (95% CI)	*p* value
pCR vs. no pCR	1.032 (0.096–1.069)	0.084
Age		0.002
40–64 vs. < 40	0.911 (0.861–0.964)	0.001
> 64 vs. < 40	0.932 (0.892–0.974)	0.002
Race		< 0.001
Black vs. White	0.797 (0.588–1.080)	0.144
Indian vs. White	0.849 (0.625–1.153)	0.295
Asian vs. White	0.786 (0.551–1.121)	0.183
Hispanic vs. White	0.871 (0.640–1.184)	0.377
Unknown vs. White	0.935 (0.689–1.269)	0.665
Pathological T stage		0.409
T1 vs. T0	1.486 (0.642–3.438)	0.355
T2 vs. T0	1.497 (0.670–3.342)	0.325
T3 vs. T0	1.554 (0.698–3.459)	0.281
T4 vs. T0	1.545 (0.693–3.444)	0.287
Tis vs. T0	1.427 (0.641–3.177)	0.384
Tx vs. T0	0.763 (0.267–2.181)	0.614
Any T mets vs. T0	1.500 (0.590–3.813)	0.395
Pathological N stage		0.549
N1 vs. N0	1.373 (0.462–4.074)	0.568
N2 vs. N0	1.399 (0.472–4.141)	0.545
N3 vs. N0	1.384 (0.468–4.090)	0.557
Nx vs. N0	1.099 (0.357–3.377)	0.870
Pathological M stage		0.986
M1 vs. M0	1.006 (0.489–2.072)	0.986
Stage		0.040
IIA vs. I	0.656 (0.171–2.511)	0.538
IIB vs. I	0.619 (0.163–2.357)	0.482
IIIA vs. I	0.552 (0.145–2.099)	0.384
IIIB vs. I	0.513 (0.135–1.949)	0.327
IIIC vs. I	0.508 (0.132–1.958)	0.325
IIINOS vs. I	0.696 (0.178–2.718)	0.602
IV vs. I	0.600 (0.203–1.776)	0.356
UNK stage vs. I	0.625 (0.158–2.468)	0.502
Subtype		< 0.001
HR+/HER2− vs. HR+/HER2+	1.188 (1.134–1.246)	< 0.001
HR−/HER2+ vs. HR+/HER2+	0.964 (0.923–1.007)	0.102
HR−/HER2− vs. HR+/HER2+	1.139 (1.077–1.205)	< 0.001

**TABLE 5:** Multivariate Cox regression analysis after PSM.

	HR (95% CI)	*p* value
Male vs. female	0.504 (0.117–2.173)	0.358
pCR vs. no pCR	7.684 (1.653–35.707)	0.009
Pathological N stage		0.146
N1 vs. N0	1.348 (0.597–3.044)	0.472
N2 vs. N0	1.768 (0.781–4.001)	0.172
N3 vs. N0	2.558 (0.997–6.560)	0.051

### 3.5. Analysis of Subtypes in MBC Patients With NAC

Among the four tumor subtypes of MBC treated with NAC, OS was significantly worse with NAC compared to adjuvant chemotherapy in both HR+/HER2− (HR = 1.363; 95% CI: 1.129–1.646; *p* = 0.001) and HR+/HER2+ (HR = 1.951; 95% CI: 1.449–2.627; *p* < 0.001) subtypes. However, no statistically significant differences were observed in HR−/HER2+ (HR = 2.296; 95% CI: 0.807–6.538; *p* = 0.119) and HR−/HER2− subtypes (HR = 1.758; 95% CI: 0.847–3.649; *p* = 0.130) (Figures 3A–D).

FIGURE 3Prognosis of NAC and adjuvant chemotherapy in MBC by tumor subtype. (A) HR+/HER2−. (B) HR+/HER2+. (C) HR−/HER2+. (D) HR−/HER2−.

**Figure:**
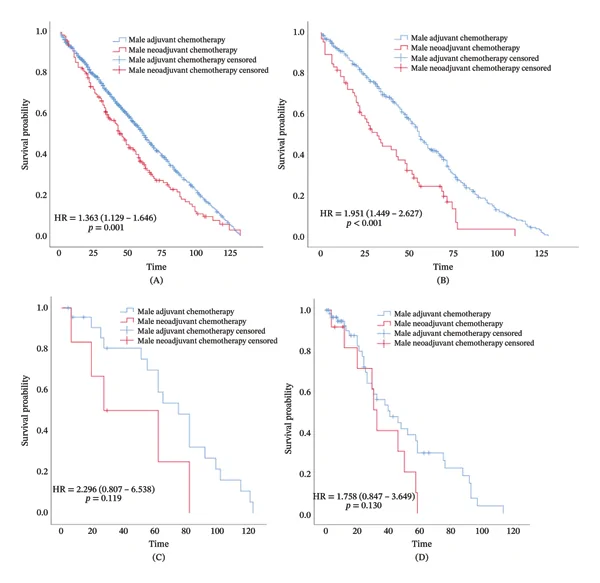

**Figure:**
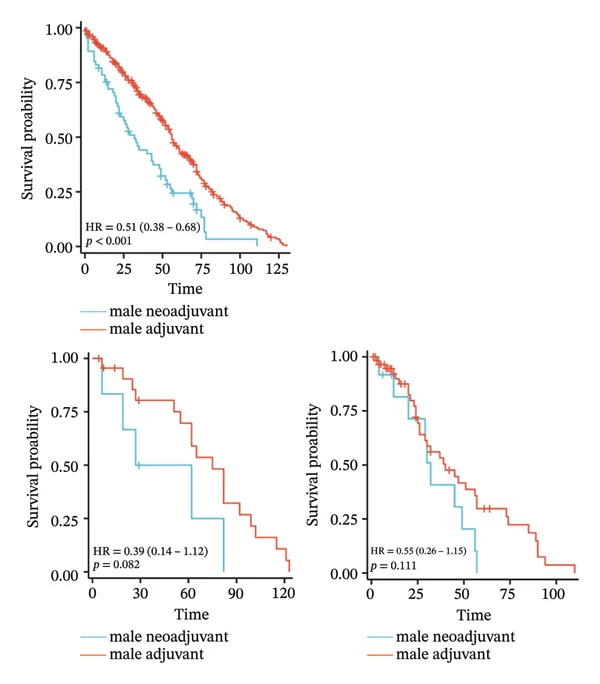

## 4. Discussion

Unlike female breast cancer, MBC is rare and remains inadequately studied. The efficacy and prognostic impact of neoadjuvant therapy for MBC are largely unknown. Therefore, we designed this study to evaluate the efficacy of NAC in MBC according to tumor subtype. The overall pCR rate to NAC in MBC was relatively low (*n* = 25, 9.6%); however, NAC demonstrated potential benefit in specific subtypes, such as triple‐negative breast cancer (TNBC) and HER2‐positive breast cancer.

Our study revealed that the overall pCR rate in male patients (9.6%) was significantly lower than that observed in female patients (29.3%). This finding is consistent with previous literature suggesting biological differences between male and female breast cancer [6]. Several studies have reported that MBC exhibits distinct molecular characteristics, including higher rates of HR positivity and lower HER2 expression compared to female breast cancer [7, 8]. These differences may contribute to differential responses to chemotherapy.

As observed in the present analysis, patients treated with adjuvant chemotherapy exhibited better OS than those receiving NAC. However, this finding should be interpreted with considerable caution. Clinically, patients selected for NAC are more likely to have locally advanced disease, larger tumor size, or lymph node involvement at initial diagnosis, leading to substantial baseline imbalance between the two treatment cohorts. Although multivariate Cox regression was used to adjust for available clinicopathological covariates, residual confounding originating from unmeasured or incompletely balanced disease characteristics could not be fully eliminated. Therefore, the survival difference between the two groups cannot be simply regarded as a direct treatment effect. Future investigations with rigorous baseline balancing are warranted to clarify whether the disparity in survival truly reflects different efficacy of treatment modalities.

Subtype‐specific analysis revealed important patterns. The HR+/HER2− subtype, which represented the largest proportion of MBC patients in our cohort, demonstrated the lowest pCR rate (5.5%). This finding suggests that HR‐positive MBC may be inherently less responsive to chemotherapy, a phenomenon also observed in female breast cancer [6]. In contrast, the HR−/HER2+ and triple‐negative subtypes showed higher pCR rates (50.0% and 41.7%, respectively), although the small sample sizes in these groups limit definitive conclusions.

Interestingly, our survival analysis revealed that achieving pCR was not significantly associated with improved OS in MBC patients (*p* = 0.357). This contrasts with extensive data in female breast cancer demonstrating that pCR is a strong prognostic indicator for improved survival outcomes [5]. Several factors may explain this discrepancy. First, the biological heterogeneity of MBC may differ from female breast cancer, potentially affecting the prognostic significance of pCR. Second, the small sample size and relatively short follow‐up period in MBC patients may have limited our ability to detect survival differences. Third, the tumor microenvironment in MBC may differ substantially from that in females, as suggested by recent single‐cell transcriptomic analyses [9, 10].

Furthermore, our comparison of NAC versus adjuvant chemotherapy in MBC demonstrated that adjuvant chemotherapy was associated with significantly better OS (*p* < 0.001). This finding persisted in subtype‐specific analyses for HR+/HER2− and HR+/HER2+ tumors. However, these results cannot suggest that adjuvant chemotherapy is the preferred treatment approach for the majority of MBC cases. Clinically, patients selected for NAC typically present with more advanced local disease, larger primary tumor burden, and/or greater lymph node involvement at diagnosis. Consequently, the observed survival difference should not be considered a direct reflection of treatment efficacy. Future studies incorporating rigorous baseline matching or adjustment are needed to determine whether the survival disparity indeed represents differential effectiveness of the treatment modalities.

Several limitations of our study warrant consideration. First, the SEER database lacks detailed information on specific chemotherapy regimens, treatment compliance, and molecular markers beyond basic receptor status. For example, this study covered a 20‐year time frame (2000–2020), during which breast cancer management underwent dramatic changes, especially following the broad adoption of HER2‐targeted therapies. Owing to the absence of detailed records on targeted treatment administration in the SEER database, reliable grouping by treatment era could not be achieved. Second, the retrospective nature of the study introduces potential selection bias, as the decision to administer NAC versus adjuvant chemotherapy was likely influenced by clinical factors not captured in the database. Moreover, the definition of pCR relied on ypT and ypN stage extracted from SEER records. Standardized coding for postneoadjuvant pathological staging was not consistently available from 2000 to 2020 in the SEER registry. Variations in registration practices over time may lead to potential misclassification of pCR status. Third, the relatively small sample sizes in certain tumor subtypes (particularly HR−/HER2+ and TNBC) limited statistical power and may have affected the significance of our findings. Fourth, the study analyzed data only through 2020, potentially missing recent advances in targeted therapies such as antibody‐drug conjugates (ADCs) and immunotherapy [11–13].

To our knowledge, this represents the most recent (updated through 2020) population‐based study reporting tumor subtypes among MBC patients treated with NAC. Our study demonstrates that men receiving NAC for breast cancer achieved significantly lower pCR rates compared to those receiving adjuvant chemotherapy. These results suggest that HR+/HER2− and HR+/HER2+ MBC subtypes may exhibit greater resistance to NAC. Importantly, we identified that pCR was not prognostic for improved survival in MBC. Therefore, routine use of NAC for MBC may not be necessary. Future prospective studies should investigate alternative treatment strategies specifically designed for the unique biological characteristics of MBC.

## Funding

This work was supported by the “Leading Goose” R&D Program of Zhejiang Province (2026C02A1111).

## Ethics Statement

The authors have nothing to report.

## Conflicts of Interest

The authors declare no conflicts of interest.

## Data Availability

Data sharing is not applicable to this article as no datasets were generated or analyzed during the current study.
